# Collagen II and decellularized hyaline cartilage scaffolds derived from bovine trachea differentially promote chondrogenic differentiation of mesenchymal stem cells and decrease secretion of angiogenic factors

**DOI:** 10.1007/s00441-025-04027-4

**Published:** 2025-12-17

**Authors:** Adriana M. Flórez, Ronald A. Jiménez, María A. Torres, Mara E. M. Braga, Herminio C. de Sousa, Marta R. Fontanilla

**Affiliations:** 1 Tissue Engineering Group, Departamento de Farmacia, Facultad de Ciencias, Universidad Nacional de Colombia, Av. Carrera 30 # 45-10, Bogotá D.C., Colombia; 2 Department of Chemical Engineering, University of Coimbra, CIEPQPF, Pólo II – Pinhal de Marrocos, Rua Sílvio Lima, 3030-790 Coimbra, Portugal; 3 Biomedical Sciences Group, Department of Medicine, Universidad Antonio Nariño, Carrera 1 # 47ª–15, Bogotá D.C., Colombia; 4 Department of Chemistry, Pharmaceutical Chemistry Program, Universidad El Bosque, Ak. 9# 131ª -20, Bogotá D.C., Colombia

**Keywords:** Bovine trachea, Collagen II scaffolds, Decellularized hyaline cartilage scaffolds, Chondrogenic differentiation, Angiogenic factor quantification

## Abstract

Collagen II scaffolds and decellularized cartilage are used for tissue engineering; however, there are no studies that compare their properties. To this aim, this study produced and characterized collagen II and decellularized cartilage scaffolds made from bovine trachea and evaluated the influence of the culture medium on the tissue type synthesized by human bone marrow mesenchymal stem cells (hBMSC) and human adipose mesenchymal stem cells (hASC) cultured on the scaffolds. Three angiogenic factors secreted by these cell cultures were also quantified. Decellularized hyaline cartilage had lower concentrations of collagen II and higher concentrations of GAG than collagen II scaffolds. The porosity, pore size, and fluid sorption capacity of the collagen scaffolds were greater than those of decellularized hyaline cartilage. Both scaffolds were hydrophilic, and their surfaces were negatively charged. The enzymatic degradation and Young’s and compression moduli of decellularized cartilage were higher than those of collagen II scaffolds. hBMSC and hASC cultured on collagen II and decellularized cartilage scaffolds with chondrogenic differentiation medium synthesized different percentages of the tissue types that made up the extracellular matrix. hBMSC on decellularized hyaline cartilage produced mainly hyaline cartilage-like tissue, whereas hASC had more immature transitional tissue. When cells were seeded onto collagen II scaffolds, transitional and fibrous tissue prevailed over hyaline tissue. Our data demonstrated that stem cell chondrogenesis in vitro was more favored by decellularized hyaline cartilage than by collagen II scaffolds, and that the concentration of angiopoietin-1, VEGF and bFGF decreased with increasing hyaline tissue formation.

## Introduction

Biocompatible scaffolds provide a provisional extracellular matrix where cells can adhere, migrate, proliferate, and differentiate toward the chondrogenic lineage. Therefore, collagen II scaffolds and decellularized cartilage matrix have been used in cartilage tissue engineering to repair damaged articular cartilage (Zhao et al. 2021).

The first scaffolds used in articular cartilage tissue engineering were made from collagen I or collagen I/III (Liu et al. 2025). However, when chondrocytes adhere to collagen I and collagen I/III fibers, they acquire a less spherical morphology than when seeded on collagen II scaffolds (Hu et al. 2024; Wu et al. 2021). On the other hand, collagen II isolated from animal sources can retain the characteristics of the native protein despite the variability of the raw materials (Wu et al. 2021). Hence, collagen II has been used for the preparation of hydrogels and scaffolds that in vitro and in vivo induce chondrogenic differentiation of mesenchymal stem cells (MSCs), preserve the differentiated phenotype of cultured chondrocytes and maintain the ability of cells to synthesize components of the cartilage extracellular matrix (Wong et al. 2018; Kilmer et al. 2020).

Decellularized cartilage has been used to treat joint injuries because the removal of cellular and extracellular matrix antigens makes it suitable for promoting the repair of damaged cartilage (Xia et al. 2019). Scaffolds derived from decellularized cartilage promote cell adhesion and chondrogenesis of chondrocytes and mesenchymal stem cells (Li et al. 2019a, b; Yin et al. 2018). Despite this, there are no studies evaluating and comparing the microstructural, physicochemical, mechanical, and biological properties of scaffolds produced with collagen II and decellularized hyaline cartilage, both isolated from bovine trachea, or their in vitro chondrogenic potential.

As reviewed (Zhang et al. 2022), common sources of cartilage extracellular matrix include human joint (Schneider et al. 2016), bovine joint (Ghassemi et al. 2017), porcine joint (Cheng et al. 2009), porcine ear (Gong et al. 2011), and porcine meniscus (Chen et al. 2015).

Manufacturing yields are low using these small-sized tissue sources; therefore, exploring larger sized sources such as trachea could help improve the cost-effectiveness of tissue-engineered cartilage scaffold production. While bovine tracheal tissue has been used to isolate proteoglycans and collagen II (Heinegård 1972; Wu et al. 2021), this tissue has not been used as a source of decellularized cartilage matrix for joint tissue engineering applications.

In this work, collagen II (Col II) and decellularized cartilage (DHC) scaffolds were produced from bovine trachea. Some microstructural (pore size and porosity), physicochemical (FTIR, quantification of hydroxyproline and glycosaminoglycans, liquid sorption capacity, contact angle, zeta potential and enzymatic degradation), and mechanical (Young’s modulus and compression modulus) properties of the Col II and DHC scaffolds were studied and compared. The cytotoxicity of the scaffolds and the proliferation of human adipose mesenchymal stem cells (hASC) and human bone marrow mesenchymal stem cells (hBMSC) seeded on them were also evaluated.

The organization of the tissue synthetized by hBMSC and hASC cultured on the scaffolds was also analyzed, since the influence of the culture medium on the tissue synthesized by human mesenchymal cells (hMSC) when cultured in different substrates is known (Zhao et al. 2022). To this end, comprehensive histological, immunohistochemical, and histomorphometric analyses were performed to compare the tissues synthesized by these cultures and to evaluate whether the differences in the microstructural, physicochemical, and mechanical properties of both scaffolds influenced the tissue formed by these cells.

Chondrocyte phenotype stability is a key factor to consider when proposing scaffolds for cartilage tissue engineering. Published work has shown that culturing MSCs on cartilage extracellular matrix hydrogels decreases secretion of angiogenic factors (Burnsed et al. 2016), and anti-angiogenic strategies have been proposed to develop products for cartilage repair (Pei et al. 2022). Therefore, the secretion of three angiogenic factors (angiopoietin-1, VEGF and bFGF) by hBMSC and hASC cultured on Col II or DHC scaffolds with or without chondrogenic differentiation medium was quantified and compared.

## Experimental

### Reagents

Ethanol 96%, sodium chloride, sodium hydroxide, and acetic acid glacial were provided by Merck (Germany). Trypsin, EDTA, collagenase I, collagenase II, pepsin, MTT (3-(4,5-Dimethylthiazol-2-yl)−2,5-Diphenyltetrazolium Bromide), StemPro™ Chondrogenesis Differentiation Kit, Dulbecco’s Modified Eagle Medium (DMEM), DMEM-Advanced medium, fetal bovine serum (FBS), antibiotic–antimycotic (100 ×), MEM vitamin solution (100 ×), non-essential amino acid (100 ×) and sodium pyruvate (1 ×) solutions were provided by Thermo Fisher Scientific (Waltham, MA, USA). Triton X-100, 3,3’-diamine benzidine, sodium dimethyl sulfoxide (DMSO), phosphate-buffered saline (PBS), alcian blue, hematoxylin and eosin, picrosirius red, safranine O, 1,9-dimethyl-methyene-blue (DMMB), Hydroxyproline Assay Kit were purchased from Sigma-Aldrich Chemical Co (St. Louis, MO, USA). The mouse monoclonal anti α-Gal epitope (M86) antibody was provided by Enzo (Farmingdale, NY, USA). Rabbit polyclonal anti-mouse collagen I antibody (PA5-95,137), mouse monoclonal anti-collagen II antibody (2B1.5, MA5-12,789), aggrecan polyclonal anti-human antibody (PA5-80,294) were provided by Thermo Fisher Scientific (Waltham, MA, USA). Goat anti-rabbit IgG (H + L) highly cross-adsorbed secondary antibody, alexa fluor plus 488, goat anti-mouse IgG (H + L) highly cross-adsorbed secondary antibody, alexa fluor plus 488, goat anti-rabbit IgG (H + L) highly cross-adsorbed secondary antibody, alexa fluor plus 564 were also provided by Thermo Fisher Scientific (Waltham, MA, USA). Genipin was purchased from Challenge Bioproducts Co (Touliu, Taiwan), the Quick-DNA™ Universal Kit by Zymo Research (Orange, CA, USA), Poietics™ human mesenchymal stem cells (hBMSC) and media were provided by Lonza (Walkersville, MD, USA) and the Magnetic Luminex® Assay was provided by R&D Systems (Minneapolis, MN, USA). NCTC clone 929 [L cell, L-929, derivative of Strain L] CCL-1™ was acquired from the American Type Culture Collection (ATCC) (Manassas, VA, USA). The bovine trachea was purchased from the San Martín packing plant (Bogotá, Colombia). Type I water was used whenever necessary.

### Preparation of Col II scaffolds

Collagen II was isolated from the clean bovine trachea. For this, clean bovine trachea fragments were mixed with trypsin (0.02% w/v, 4 °C, 24 h) in PBS (pH 7.2). The resulting suspension was homogenized (10.000 rpm, 20 min, Ultra-Turrax®, IKA Works, China), and centrifuged at 420 × *g* (Thermo Fisher, USA) for 30 min. The supernatant was removed; the pellet was rinsed with deionized water and re-suspended in acetic acid solution (0.5-M, pH 2.4), incubated (60 rpm, 4 °C, 32 h), and centrifuged (400 × *g*, 30 min). The supernatant was collected, and collagen II was salted out with NaCl (5-M). The resulting pellet was washed with deionized water and centrifuged (420 × *g*, 30 min), the supernatant was collected and resuspended in acetic acid solution (0.5-M, pH 2.4). The collagen II dispersion (5 mg/g) obtained was poured into molds, frozen (− 20 °C), and lyophilized for 48 h (Virtis, SP Industries, USA). The resulting porous scaffolds (10 × 10 cm) were cross-linked with genipin (0.05%, 48 h), washed with ethanol and water, frozen (− 20 °C), and lyophilized again for 48 h.

### Preparation of DHC scaffolds

The bovine trachea was cleaned to remove unwanted tissue and cut with a microtome (SLEE medical, Germany) to obtain slides 300 µm thick. Trachea slides were immersed in water for 24 h, incubated with NaOH (1-N, pH 13, 3 h) and rinsed with water. Thereafter, they were incubated (37 °C, 4 h) with trypsin/EDTA (0.25%/0.02%), rinsed with water, incubated with Triton X-100 (1%, 24 h) at room temperature, and washed with water. Finally, tissue slides were immersed in NaOH (1-N, pH 13, 3 h), washed with water, frozen (–20 °C), and lyophilized (48 h).

#### Histological and immunohistochemical analyses of DHC scaffolds

These analyses were carried out to demonstrate the removal of bovine chondrocytes, glycosaminoglycans (GAGs), collagen, and α-gal epitope from DHC scaffolds. The native cartilage and DHC samples (5 mm [∅], 0.3 mm [H]) were fixed with 10% formaldehyde in PBS (pH 7.4, 4 °C, 24 h), washed with PBS, embedded in paraffin, and sectioned. Chondrocytes and GAGs were assessed by hematoxylin–eosin (H&E) and alcian blue staining, respectively. The presence and orientation of collagen fibers were evaluated with picrosirius red staining. For the immunohistochemical detection of α-gal epitope (Galα1-3Galβ1-4GlcNAc-R), slices were placed in a solution of hydrogen peroxide (3%) in tris-saline buffer (TBS, pH 7.4) for 10 min and washed twice with PBS. After washing, samples were blocked for 10 min at room temperature with TBS containing 5% milk and incubated for 1 h with α-gal monoclonal antibody diluted 1:5 in TBS. Samples were rinsed twice with PBS, incubated with a secondary antibody for 15 min, rinsed with PBS, and revealed with 3,3-diaminobenzidine (DAB) (0.05%) and hydrogen peroxide (0.01%) in Tris–HCl buffer.

#### Biochemical evaluation of DHC scaffolds

DNA was quantified in DHC scaffolds to confirm decellularization; in this assay, native cartilage was included as a negative control of decellularization. DNA was isolated from native cartilage and DHC scaffolds using the Quick-DNA™ Universal Kit following the manufacturer’s instructions. The efficiency of the decellularization was evaluated by reading the concentration of DNA in native cartilage and DHC scaffolds.

The collagen content of native cartilage and DHC scaffolds was determined by measuring hydroxyproline content (Cissell et al. 2017). For this, the hydroxyproline residues were oxidized with chloramine T and colorimetrically detected with p-dimethylaminobenzaldehyde (DMAB). The absorbance of the samples was read at 560 nm with a TRIAD Multimode Detector (Dynex, Elkin, NC, USA) and the values obtained were interpolated into a calibration curve (R^2^ = 0.995) made by measuring the absorbance of serial dilutions prepared by degrading known amounts of native cartilage with collagenase II.

The GAGs content of native cartilage and DHC scaffolds was determined colorimetrically following a described dimethyl methylene blue (DMMB) procedure (Farndale et al. 1986). The absorbances of the samples were measured at 595 nm and the resulting values were interpolated into a calibration curve (R^2^ = 0.991) made by measuring the absorbances of serial dilutions prepared from samples of enzymatically degraded native cartilage. All experiments described above were repeated four times.

### Microstructural characterization of the scaffolds

#### Pore size and porosity

Col II and DHC scaffolds of the same size were analyzed by scanning electron microscopy (SEM) using a QUANTA 250 SEM (FEITM, Hillsboro, OR, USA). Images captured with the xT Microscope Control software (version 2.01) were used to calculate the mean pore size by measuring the major and minor axes of the ellipses that best fitted each pore using the ImageJ 1.39 k software (Wayne Rasband, NIH, USA). These values were multiplied by 1.5 to consider the pores that were not sectioned through their maximal cross-section. A total of 50 pores per scaffold were analyzed. Pore size was calculated using the following equation:

1 $$PS=1.5\times 2X\sqrt{\frac{{a}^{2}+{b}^{2}}{2}}$$

Where a is the major axis and b is the minor axis.

A published liquid displacement method was modified to assess the porosity of the scaffolds (Ho and Hutmacher 2006). Samples of Col II and DHC scaffolds of the same size were weighed (W_1_), immersed in 2 mL of 1X PBS, degassed under vacuum (10 min), and kept at 4 °C for 24 h. Samples were removed, placed on filter paper to remove the excess of PBS, weighed (W_2_), and placed in a dry pycnometer of known weight (W_3_). The pycnometer was filled with PBS until its volume (25 mL) was completed and weighed (W_4_). Assays were carried out four times. Porosity was calculated using W_1_, W_2_, W_3_, and W_4_ and the density (ρ) of PBS (1.023 g/mL) as follows:

2 $$P\left(\%\right)=1-\left(\frac{\rho wet\;scaffold}{\rho collagen\;fibers}\right)\times100$$

Where ρ_wet scaffold_ = (W2)/[(25 mL-(W4-W3-W2)/(1.023 g/mL)], and ρ_collagen fibers_ = (W1)/[((1.023 g/mL × 25 mL) − W4 + W3 + W1)/(1.023 g/mL)].

### Physicochemical characterization of the scaffolds

#### Fourier transform infrared spectrometry (FTIR)

This analysis was performed to identify Col II isolated from the bovine trachea and the presence of this protein in DHC scaffolds. Samples of Col II and DHC scaffolds were analyzed with a FTIR spectrophotometer (IRAffinity 1S, Shimadzu, Japan) that incorporates an attenuated total reflection cell. Spectra were obtained in a range between 4000 and 500 cm^−1^ with a resolution of 4 cm^−1^ and 64 scans/sample.

#### Biochemical evaluation

Differences in concentration of collagen II and GAGs in Col II and DHC scaffolds were determined as described previously (the “Biochemical evaluation of DHC scaffolds” section). Unlike the biochemical analysis performed during the evaluation of the decellularization, for these quantifications the measured absorbance values were interpolated into calibration curves made with known concentrations of hydroxyproline and chondroitin sulfate. The experiments were repeated four times.

#### Liquid sorption capacity (LSC), contact angle (CA), and zeta potential (ζ)

Liquid sorption capacity (LSC) was determined gravimetrically. Samples of Col II and DHC scaffolds of the same size were weighed (W_1_), covered with DMEM supplemented [FBS (10%), penicillin (100 units/mL), streptomycin (100 μg/mL), MEM vitamin solution (1%), sodium pyruvate (1%), non-essential amino acid (1%)] to mimic physiological conditions, and incubated (37 °C). The samples were taken with tweezers and placed on filter paper to remove the excess of liquid and weighed (W_2_). Experiments were repeated three times and scaffolds’ LSC was calculated at 24 and 48 h according to the following equation:

3 $$LSC (\%)=\left(\frac{\text{W}2-\text{W}1}{\text{W}1}\right)\times 100$$

The dynamic contact angle (CA) of the scaffolds was measured at the liquid–solid interface using the captive bubble method with an OCA 20 device (DataPhysics Instruments, Filderstadt, Germany). Samples of Col II and DHC scaffolds of the same size were hydrated in supplemented DMEM for 24 h at room temperature. After that, the scaffolds were fixed in the device holder, immersed in supplemented DMEM, and a bubble was formed by injecting air (6 μL) beneath the surface of the scaffolds. For 120 s a total of 200 digital images of the bubble formed were captured. The experiments were repeated four times.

The electric surface potential (zeta potential) of Col II and DHC scaffolds in PBS was measured with a Zetasizer Nano ZS (Malvern Instruments, Malvern, Worcestershire, UK). The scaffolds (0.2 g) were placed in 2 mL of PBS (20 mM, pH 7.4) for 12 h, and processed with an Ultra-Turrax homogenizer system (IKA 3737001 T10 basic, Cole-Palmer, UK) at 3000 rpm for 10 min. Five measurements were made per scaffold (100 runs each at 25 °C).

#### Enzymatic degradation

Scaffolds degradation was evaluated by enzymatic digestion. Dry samples (2 mg) of Col II and DHC scaffolds were weighed (W_1_) and incubated (37 °C, 80 rpm) with 1 mL (5UI/mL PBS) of collagenase II for 2, 4, 6, and 8 h. At each time samples were centrifuged (600 × *g*, 5 min), washed with deionized water, lyophilized, and weighed (W_2_). Experiments were repeated four times. The percentage of enzymatic degradation was calculated using the following equation:

4 $$D (\%)=\left(\frac{\text{W}1-\text{W}2}{\text{W}1}\right)\times 100$$

### Mechanical characterization of the scaffolds

#### Traction assay

The uniaxial traction assay was performed in a micro material testing machine (MMT, Shimadzu, MD, USA), equipped with a 101 N load cell, at a speed of 1.0 mm/s until the scaffolds ruptured. Samples of Col II and DHC scaffolds of the same size were hydrated in supplemented DMEM for 24 h before the assay. The Young’s modulus of the scaffolds was calculated by measuring the slope of the linear zone of the stress–strain curves. It is presented as the average of four measurements for each scaffold.

#### Unconfined compression assay

Samples of Col II and DHC scaffolds of the same size were tested in unconfined uniaxial compression using a texturometer (LAMY TX-700), equipped with a 50 N load cell at a speed of 0.1 mm/s, until 50% of maximum deformation was reached. Samples were hydrated in supplemented DMEM for 24 h before the assay. The compressive modulus of the scaffolds was calculated from the slope of the stress–strain curves over a strain range of 1% to 5%. The slope of the straight line that best fitted was taken as the compressive modulus, which is presented as the average of four measurements for each scaffold.

### Bioactivity of the scaffolds

#### Cytotoxicity assay

Cytotoxicity of Col II and DHC scaffolds were assessed following ISO 10993–5 (ISO 2009). For this, NCTC clone 929 fibroblasts were seeded in 96-well plates (1 × 10^4^ cells/100 µL medium) and incubated (37 °C, 5% CO_2_, 24 h) with supplemented DMEM. The culture medium was replaced by an extraction medium, which was obtained by incubating the sterile scaffolds with DMEM (37 °C, 5% CO_2_, 24 h). Cell cultures incubated with 25% DMSO in DMEM or incubated with silicone (0.2 g/mL DMEM) were used as positive and negative controls, respectively. After removing the tested media, 50 µL of MTT (1 mg/mL) was added and samples were incubated under the conditions described above. After MTT was discarded, DMSO (100 µL) was added, and the absorbance of the resulting solution was measured at 570 nm. These experiments were repeated three times and the results are presented as the average of the three experiments.

#### Isolation and culture of MSCs

Human adipose mesenchymal stem cells (hASC) were isolated by modifying a described procedure (Araña et al. 2013). A sample (3 g) of adipose tissue isolated from a healthy young donor liposuction with signed informed consent, was incubated with collagenase I (2 mg/mL DMEM) for 1 h at 37 °C. Subsequently, supplemented DMEM was added to inactivate the enzyme and the cell suspension was filtered with a cell strainer (70 µm). The resulting filtrate was centrifuged (300 × *g*, 5 min) and the pellet was resuspended in supplemented DMEM. Cells (5.0 × 10^3^ cells/cm^2^) were seeded in T-75 flasks and incubated (37 °C, 5% CO_2_) changing medium every 3 days until the culture reached 90% confluence. The primary culture was sub-cultured using the same medium. Cells from the fourth passage were used for in vitro assays.

To obtain the hBMSC cultures, Poietics™ human mesenchymal stem cells (5.0 × 10^3^ cells/cm^2^) were seeded in T-75 flasks and incubated (37 °C, 5% CO_2_) with supplemented DMEM. The culture medium was changed every three days until a 90% cell confluence was reached. The primary culture was then sub-cultured, and cells from the fourth passage were used for in vitro assays. Results are presented as the average of three experiments.

#### MSC proliferation assay

Sterile samples (19.6 mm^2^) of Col II and DHC scaffolds were seeded with hASC or hBMSC (5.0 × 10^4^ cells/scaffold) suspended in 20 µL of supplemented DMEM. After incubating for 2 h to allow the cells to attach to the scaffolds, cultures were covered with 1 mL of supplemented DMEM and incubated (37 °C, 5% CO_2_) for 1, 3, 7, and 14 days. At each time the medium was removed, replaced by 500 µL of MTT (1 mg/mL) solution, and incubated (37 °C, 5% CO_2_) for 4 h. The formazan crystals formed were solubilized with 1 mL of DMSO and the absorbance of the resulting solution was measured at 570 nm (TRIAD Multimode Detector, Dynex). The absorbance of each sample was interpolated into a calibration curve that was made with a known number of cells (hASC or hBMSC). The experiments were repeated three times.

#### Histological, immunohistochemical and histomorphometric analyses

For the histological analysis, sterile samples of Col II and DHC scaffolds of the same size were seeded with hASC or hBMSC (1.5 × 10^5^ cells/20 µL medium) and incubated (37 °C, 5% CO_2_) in supplemented DMEM medium or in chondrogenic differentiation medium for 28 days changing the medium every other day. On the last day, the medium was removed, and the samples were washed with PBS, fixed with 10% formaldehyde in PBS (pH 7.4), incubated (4 °C, 24 h), washed with PBS, embedded in paraffin, sectioned, and stained with H&E or safranin-O. The stained slides were observed under a light microscope (Nikon ECLIPSE 55i, Nikon, Tokyo, Japan).

For the immunostaining, the scaffolds were dehydrated in an ascending series of ethanol (70, 80, 90, 100% v/v), washed with xylene, embedded in low melting point paraffin, and sectioned (5 μm). The tissue sections were placed on glass slides and rehydrated with 10 mM PBS. Before immunodetection of collagen II, samples were incubated (3 min, 37 °C) with pepsin (1 mg/mL) in Tris–HCl (pH 2.0). Then, all samples were blocked with 3% albumin in 10 mM PBS for 30 min at room temperature and incubated with anti-collagen I, anti-collagen II, and anti-aggrecan primary antibodies solubilized in the blocking solution. Following incubation in a humidity chamber (4 °C, 12 h), samples were washed with 1X PBS (5 min, three times), and incubated (2 h) with the secondary antibody diluted in the blocking solution. After washing with 10 mM PBS (5 min, three times), 50 μL DAPI diluted in 10 mM PBS was added to each sample and incubated. The stained slides were observed under a fluorescence microscope (Nikon ECLIPSE TS100, Tokyo, Japan).

The histomorphometric analysis was carried out on representative histological sections (*n* = 9) from 28-day cultures. Cell nucleus morphology, extracellular matrix appearance, safranin-O staining, and immunostaining for type I and type II collagen were used to classify tissue formed on Col II and DHC scaffolds seeded with hASC or hBMSC (see Table 1) (Dorotka et al. 2005). For this evaluation, the total area of each digital image and the area percentages of each type of tissue synthesized de novo were determined. Cell nucleus morphology (ε) was assessed by dividing the minor axis over the major axis of an ellipse adjusted to the shape of each nucleus (Toh et al. 2012). The NIS ELEMENTS F2.20 software (Nikon, Chiyoda-Ku, Tokyo, Japan) and ImageJ [http://imagej.nih.gov/ij/] were used for this assessment.

**Table 1:** Newly formed tissue types

Tissue types	Characteristics
Hyaline tissue	• Ground glass-like appearance of the matrix • Spherical-shape cells with rounded nuclei (ε ≥ 0.5) in lacunae • Varying intensity of staining for safranin-O and type II collagen
Transitional tissue	• Encompassed a wide range of tissue between fibrous tissue and hyaline cartilage, including fibrocartilage • Spherical-shape cells with rounded nuclei (ε ≥ 0.5) with and without lacunae • Varying intensity of staining for safranin-O and type I and II collagen
Fibrous tissue	• Extracellular matrix with fibrous appearance • Spindle-shape cells with elongated nuclei (ε < 0.5) without lacunae • No safranin-O staining

#### Quantitation of angiogenic factors

Media from hASC or hBMSC cultured on Col II and DHC scaffolds were evaluated. On day 28, the culture medium was removed, cells were washed twice with PBS, and incubated with reduced serum DMEM-Advanced medium for 3 days. Then, aliquots of culture medium were taken to quantify angiopoietin 1, VEGF and bFGF with a Magnetic Luminex® Assay (R&D Systems, Minneapolis, MN, USA) following the manufacturer’s instructions. The secretion of angiogenic factors was normalized by the number of cells per scaffold determined by the MTT cell viability assay. Assays were performed in triplicate.

### Statistical analysis

Data are shown as the mean ± the standard deviation (SD). Statistical analyses were performed using one-way analyses of variance (ANOVA) and Tukey’s *post-hoc* tests or Student’s *t*-test. Differences were considered statistically significant at *p* < 0.05. GraphPad Prism 8 Software was used to conduct all the statistical analyses.

## Results

### Decellularization of hyaline cartilage

This work combined and modified published procedures (Gong et al. 2011; Schwarz et al. 2012; Yang et al. 2010) to decellularize bovine tracheal hyaline cartilage with an affordable and fast method. Histological, immunohistochemical, and biochemical analyses of the native and DHC scaffolds were performed to assess the quality of the decellularization process. The results from these analyses are presented below.

#### Histological, immunohistochemical and biochemical analyses of DHC scaffolds

**Fig. 1:**
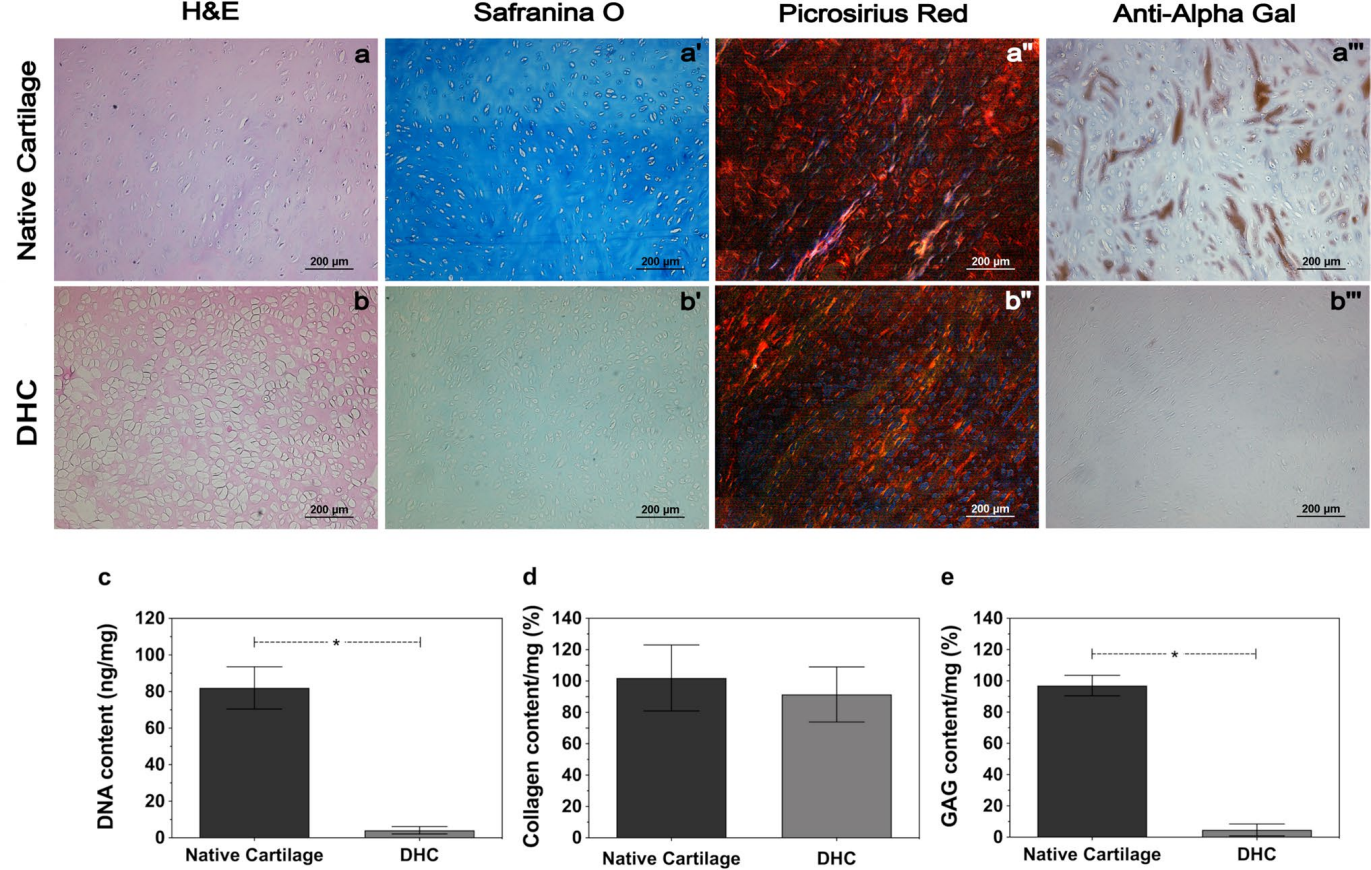
Histological, immunohistochemical and biochemical analyses of DHC scaffolds. Hematoxylin–eosin (H&E), safranin O, picrosirius red and immunostained with anti-α-gal monoclonal antibody staining of (a–a’’’) native cartilage and (b–b’’’) DHC scaffolds. Content of (c) DNA (d) collagen, and (e) GAGs in native cartilage and DHC scaffolds. Number of replicas (*n*) = 4; mean ± SD; *p < 0.05

Representative images of histological and immunohistochemical analyses are shown in Fig. 1. Hematoxylin and eosin staining showed chondrocytes in native cartilage and the absence of cells in DHC scaffolds (Fig. 1a, b). Alcian blue staining for alcianophilic GAGs was strongly positive in native cartilage and attenuated in DHC scaffolds (Fig. 1a’, b’), suggesting a decrease in GAGs content upon decellularization. Picrosirius red staining was similar in both tissues, indicating that decellularization does not significantly affect the collagen content of DHC scaffolds (Fig. 1a’’, b’’). Furthermore, the orientation of collagen fibers was preserved, as observed with polarized light microscopy. Finally, immunostaining of the α-gal epitope was positive in native cartilage and negative in decellularized samples (Fig. 1a’’’, b’’’).

The removal of cells and components of the extracellular matrix was assessed to control the quality of the decellularization process. Cell removal was determined by quantifying DNA. The DNA concentration found in native cartilage was significantly higher (82 ± 11.5 ng/mg) than that found in DHC scaffolds (4 ± 1.6 ng/mg) (Fig. 1c).

The quantification of free hydroxyproline after alkaline hydrolysis of native cartilage and DHC scaffolds was used as an indirect measure of their collagen content. The percentage of hydroxyproline in DHC scaffolds was 91.5 ± 18% of the hydroxyproline percentage found in native cartilage (Fig. 1d), confirming that decellularization does not significantly affect the collagen II content of DHC scaffolds as was established by the picrosirius red stain. Trace amounts of GAGs that remain after decellularization help cells adhere to and grow on the decellularized scaffold (Sutherland et al. 2015). Therefore, it was important to confirm, not only qualitatively but quantitatively, the content of GAGs remaining after decellularization. Figure 1e shows the GAGs percentage in DHC scaffolds, which was 4.6 ± 3.7% of that found in native cartilage indicating the presence of GAG remnants.

### Characterization of the scaffolds

#### Pore size and porosity

**Fig. 2:**
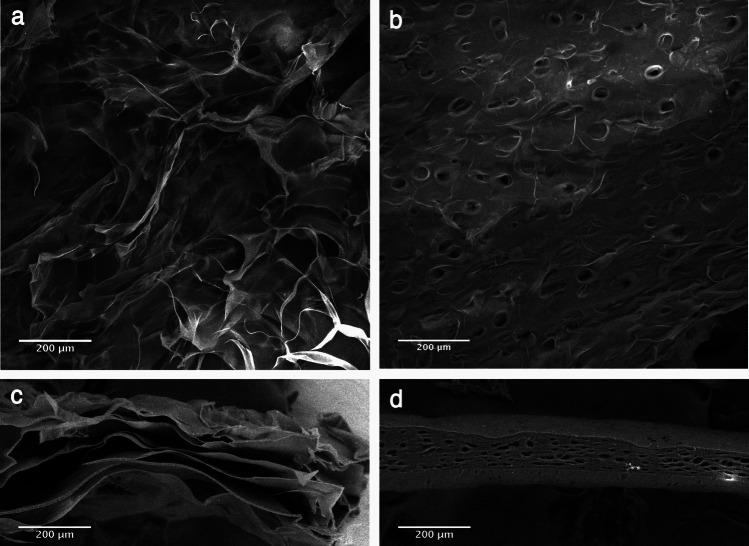
SEM evaluation of the scaffolds. Longitudinal sections of (**a)** Col II and (**b)** DHC scaffolds. Cross-sections of (**c**) Col II and (**d)** DHC scaffolds

Scanning electron microscopy was used to assess the microstructure of both types of scaffolds (Fig. 2). Longitudinal sections of the Col II scaffolds showed a microstructure with interconnected pores, apparently of different sizes (Fig. 2a), whereas longitudinal sections of DHC scaffolds showed cell-free lacunae and a well-preserved native cartilage microstructure (Fig. 2b). Cross-sections showed the multilamellar structure of Col II scaffolds (Fig. 2c) and confirmed the preservation of tissue structure and the removal of chondrocytes from the lacunae in the DHC scaffolds (Fig. 2d). The mean pore size (264.9 ± 84.1 µm) and porosity (92 ± 5.9%) of Col II scaffolds was significantly larger (*p* < 0.05) than those of the DHC scaffolds (36.6 ± 7 µm and 61.5 ± 1.8%, respectively).

#### Fourier transform infrared spectrometry (FTIR)

**Fig. 3:**
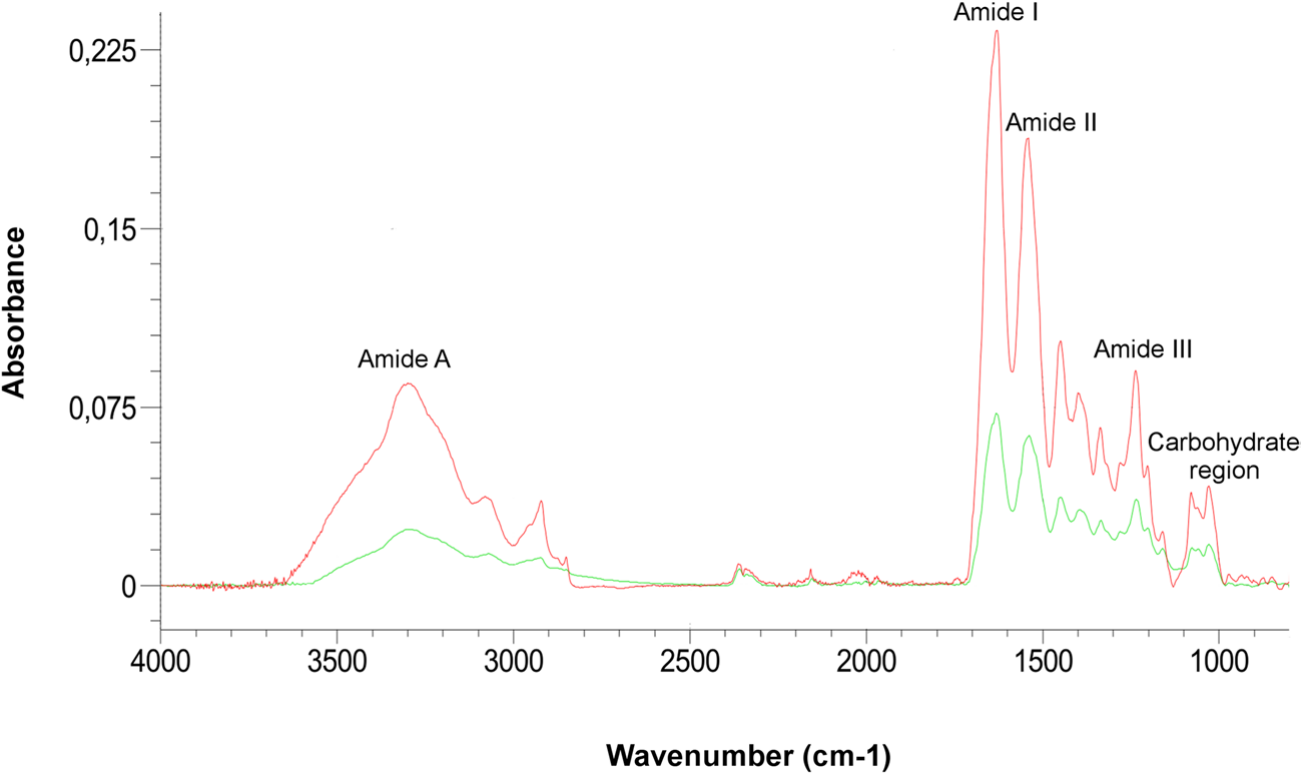
Attenuated total reflection (ATR)-Fourier Transform IR Spectroscopy (ATR-FTIR) analysis of the scaffolds. Spectra of: (red line) Col II, and (green line) DHC scaffolds

The FTIR results are shown in (Fig. 3). The spectrum of Col II scaffolds exhibited the main absorption bands of this protein: [amide-A (N–H stretch) at 3300 cm^−1^; amide-I (C = O stretch) at ≈1655 cm^−1^; amide-II (C-N stretch, N–H bend combination) at ≈1550 cm^−1^; amide-III (C-N stretch, N–H bend, C–C stretch) at ≈1250 cm^−1^]. They also showed bonding vibrations between ≈1020 and 1080 cm^−1^ probably related to the presence of GAGs. The spectrum of the DHC scaffolds showed the same absorption bands; however, their intensity was lower than that of the Col II scaffolds (Camacho et al. 2001).

#### Biochemical evaluation, enzymatic degradation, and mechanical tests

**Fig. 4:**
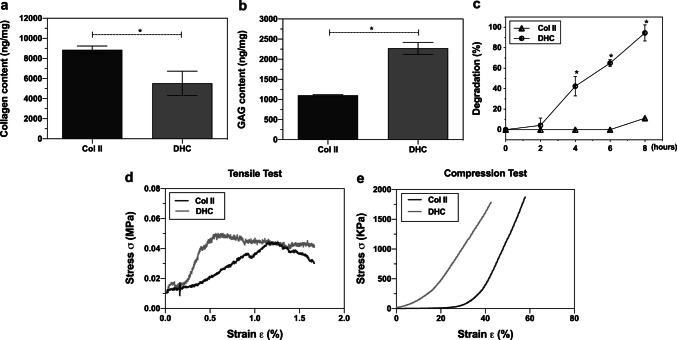
Biochemical evaluation, enzymatic degradation and mechanical tests of the Col II and DHC scaffolds. Content of (**a)** Collagen and (**b)** GAGs in the scaffolds; (**c)** enzymatic degradation; (**d)** Young’s modulus and (**e)** compression modulus. Number of replicas (*n*) = 4; mean ± SD; *p < 0.05

Once the Col II and DHC scaffolds were prepared, their content of collagen II and GAGs was quantified to confirm the qualitative findings of the ATR-FTIR analyses. For these quantifications, calibration curves were made with known concentrations of hydroxyproline and chondroitin sulfate. The Col II scaffolds presented a significantly higher (*p* < 0.05) collagen concentration (8854.86 ng/mg dry scaffold) than that of the DHC scaffolds (5521.76 ng/mg) (Fig. 4a). As expected, the concentration of GAGs in the Col II scaffolds (1101.50 ng/mg) was significantly lower than that of the DHC scaffolds (2270.67 ng/mg) (Fig. 4b).

The in vitro enzymatic degradation profile for Col II and DHC scaffolds was different, and at all times tested (4, 6, and 8 h) the degradation percentage of decellularized scaffolds was significantly higher (*p* < 0.05) than that of Col II scaffolds (Fig. 4c).

Data from the tensile and compression tests were used to calculate Young’s modulus and compression modulus, respectively. The Young’s modulus and compression modulus of DHC scaffolds (0.135 MPa and 4.010 kPa, respectively) were significantly higher than those of the Col II scaffolds (0.038 MPa and 0.185 kPa, respectively) (Fig. 4d, e).

#### Liquid sorption capacity (LSC), contact angle (CA), and zeta potential (ζ)

The in vitro interactions between scaffolds and aqueous fluids or cells are predictive of their behavior in the physiological environment (Drobota et al. 2022). The LSC, CA, and ζ potential values of Col II and DHC scaffolds found in this work are shown in Table 2. The LSC of Col II scaffolds was significantly higher (*p* < 0.05) than that of DHC scaffolds at 24 and 48 h. Furthermore, the Col II scaffolds showed a significantly lower CA (*p* < 0.05) than the DHC scaffolds; however, both CA values were less than 90°, indicating the hydrophilicity of both scaffolds. Finally, the ζ mean value of the Col II and DHC scaffolds was negative, and there were no significant differences (*p* > 0.05) between them.

**Table 2:** Liquid sorption capacity, contact angle, and zeta potential

	Liquid sorption capacity (%) 24 and 48 h	Contact angle (°)	Zeta potential (mV)
Collagen II	781.5 ± 33.9–737 ± 55.9	56.8 ± 9.7	− 8.34 ± 0.9
Decellularized hyaline cartilage	228.9 ± 65.8–233.3 ± 20	83.7 ± 5.3	− 7.37 ± 2.1

### Bioactivity of the scaffolds

#### Cytotoxicity and MSC proliferation assay

**Fig. 5:**
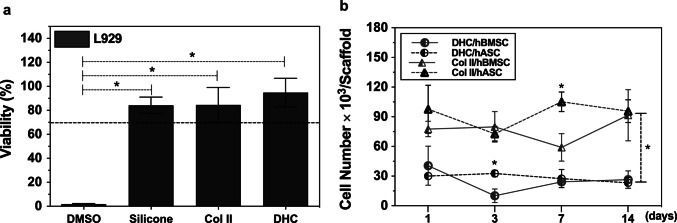
Cytotoxicity and MSC proliferation assay. (**a)** Viability percentages of L929 fibroblasts incubated with extraction media from Col II scaffolds, DHC scaffolds, negative (silicone) and positive (DMSO) controls. (**b)** Growth curves of hASC and hBMSC seeded on Col II and DHC scaffolds. Number of replicas (*n*) = 3; mean ± SD; *p < 0.05

A cytotoxicity evaluation of the scaffolds was performed to verify their safety (Fig. 5a). Exposure of L929 fibroblast cultures to the extraction medium prepared by incubating the scaffolds with DMEM resulted in viability percentages greater than 70%. A similar result was observed when incubating the cells with the negative control (silicone extraction medium). Reduction of cell viability percentages of L929 fibroblasts incubated with leachates from the negative control (silicone), Col II and DHC scaffolds was less than 30%, confirming that the scaffolds were noncytotoxic. In contrast, in cultures incubated with DMSO extraction medium (positive control) the percentage of cell viability decreased to 1.9%.

The evaluated scaffolds are designed for surgical treatments of articular cartilage injuries, where blood from the subchondral bone enters the joint. Therefore, growth curves of hASC or hBMSC seeded on Col II and DHC scaffolds were obtained by evaluating cell viability as a function of time. Overall, the number of both cell types was significantly higher (*p* < 0.05) in Col II than in DHC scaffolds. Significant differences (*p* < 0.05) in cell number were observed between the two scaffolds only on day 7 of hASC cultures and on day 3 of hBMSC cultures. On day 14, no significant differences were observed between hASCs and hBMSCs cultured on Col II scaffolds, nor between those cultured on DHC scaffolds. However, significant differences in cell proliferation were observed between the two scaffolds (Fig. 5b).

#### Histological, immunohistochemical, and histomorphometric analyses

**Fig. 6:**
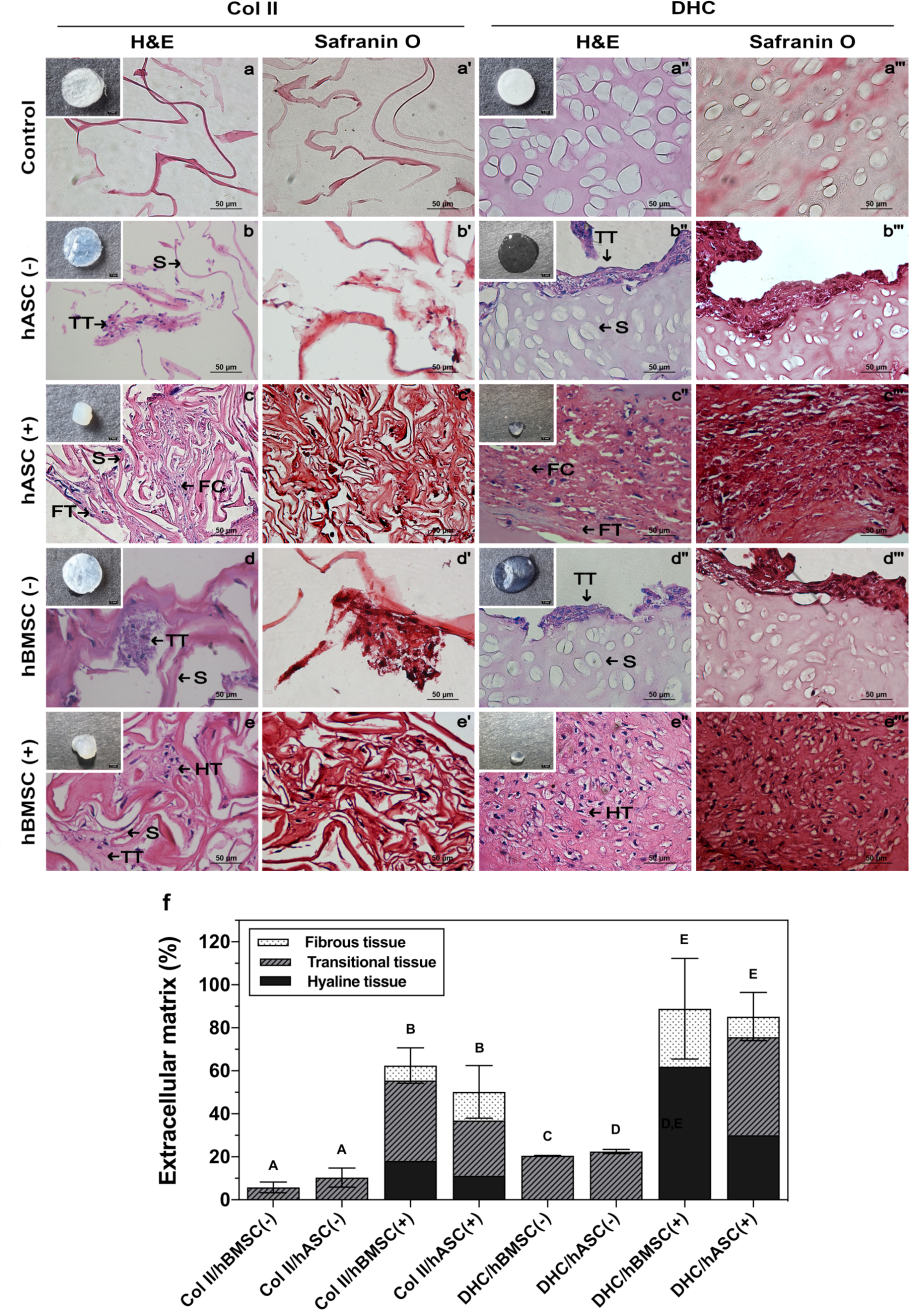
Histological and histomorphometric analyses. Figure shows hematoxylin–eosin (H&E) and safranin O staining of (a-a’, b-b’, c–c’, d-d’, e-e’) Col II and (a’’-a’’’, b’’-b’’’, c’’-c’’’, d’’-d’’’, e’’-e’’’) DHC scaffolds seeded with hASC (b-c’’’) or hBMSC (d’-e’’’) and cultured with (+) or without (-) chondrogenic differentiation medium for 28 days. The insets in the upper left corner show the appearance of cell-seeded scaffolds after incubation. Figure 6f shows the ImageJ quantification of the percentage of ECM formed in Col II and DHC scaffolds seeded with hASC or hBMSC and cultured with or without chondrogenic differentiation medium for 28 days. FC, fibrocartilage; FT, fibrous tissue; TT, transitional tissue; HT, hyaline tissue; S, scaffold. Number of replicas (*n*) = 3; mean ± SD; groups that do not share a letter are significantly different (*p* < 0.05)

Histological analysis (H&E and safranin O staining) of the mesenchymal cell–seeded scaffolds was performed to assess extracellular matrix formation (Fig. 6). The insets in the upper left corner of the H&E images show images of Col II and DHC scaffolds cultured with cells at day 28. Notably, cultures grown in the absence of chondrogenic differentiation medium did not show contraction, whereas those grown with chondrogenic differentiation medium did.

The appearance of unseeded Col II and DHC scaffolds used as controls is presented in Fig. 6a-a’’’. Representative images of hASC cultured on Col II scaffolds without differentiation medium and stained with H&E or Safranin O showed some fibers and detached transitional tissue (Fig. 6b, b’). When these cells were cultured on DHC scaffolds under the same conditions, H&E or Safranin O staining showed transitional tissue formed at the outer edges of the scaffold (Fig. 6b’’, b’’’). hASC cultured in Col II scaffolds and incubated with differentiation medium formed internal fibrocartilage zones containing small lacunae with or without cells, as well as fibrous tissue (Fig. 6c, c’). When these cells were cultured on DHC scaffolds, they were remodeled and replaced by transitional tissue composed mainly of fibrocartilage, in which lacunae with or without cells were observed (Fig. 6c’’, c’’’).

hBMSCs cultured without differentiation medium synthetized scattered islets of transitional tissue when seeded on Col II scaffolds (Fig. 6d, d’), whereas when seeded on DHC scaffolds they formed transitional tissue at the outer edges of the scaffold (Fig. 6d’’, d’’’). The effect of the differentiation medium on the tissue formed by hBMSC was remarkable, as Col II scaffolds were replaced by transitional and hyaline cartilage (Fig. 6e, e’). Most of the mature cartilage with lacunae surrounded by a territorial matrix with round nuclei was synthetized by hBMSC in the DHC scaffolds (Fig. 6e’’, e’’’).

Histological images were analyzed using ImageJ software to quantify the percentage of ECM formed in Col II and DHC scaffolds seeded with hASC or hBMSC and cultured with or without chondrogenic differentiation medium for 28 days. Only hBMSC or hASC cultured on the Col II scaffolds without chondrogenic differentiation medium produced transitional tissue, and no significant differences were observed between them (*p* > 0.05). Although fibrous, transitional, and hyaline tissue were observed when chondrogenic differentiation medium was used, there were no significant differences (*p* > 0.05) between them. In cultures grown on DHC scaffolds without differentiation medium, only transitional tissue was observed, and there were significant differences (*p* < 0.05) in the ECM percentages found. When the chondrogenic differentiation medium was used, hBMSC replaced the DHC scaffold with hyaline tissue in a percentage greater than 60%, with the remaining percentage being fibrous tissue. Under the same conditions, hASC synthetized 30% of hyaline tissue, more than 40% transitional tissue, and approximately 10% fibrous tissue (Fig. 6f).

Histomorphometric analysis confirmed the presence of different tissue types within the same scaffold. hBMSCs cultured on DHC scaffolds with chondrogenic differentiation medium synthesized a high percentage of hyaline tissue and a low percentage of fibrous tissue. Under the same culture conditions, hASCs formed a higher percentage of transitional tissue than hyaline tissue. Finally, both mesenchymal cell types cultured on Col II scaffolds with chondrogenic differentiation medium synthetized more transitional tissue than hyaline and fibrous tissue, the latter located in the periphery of the scaffolds. Overall, the percentages of synthesized extracellular matrix were higher in the presence of chondrogenic differentiation medium than when incubated with DMEM (*p* < 0.05). Hyaline tissue was only synthesized in cultures incubated with chondrogenic differentiation medium, with the highest percentage of this tissue being observed in DHC scaffolds seeded with hBMSC. The percentages of ECM, fibrous tissue (FT), transitional tissue (TT), and hyaline tissue (HT) synthesized de novo in each culture are also shown (Table 3).

**Table 3:** De novo synthesized ECM and tissue types observed in each culture

Culture ^a^	Synthesized ECM (%)	Types of tissue		
		FT (%)	TT (%)	HT (%)
Col II/hBMSC (-)	5.77 ± 2.51		5.77 ± 2.51	
Col II/hASC (-)	10.36 ± 4.47		10.36 ± 4.47	
Col II/hBMSC (+)	62.10 ± 8.24	7.02 ± 4.62	36.93 ± 14.51	18.15 ± 16.60
Col II/hASC (+)	51.87 ± 12.25	13.31 ± 10.01	25.67 ± 24.65	12.89 ± 11.37
CHD/hBMSC (-)	20.57 ± 0.02		20.57 ± 0.02	
CHD/hASC (-)	22.48 ± 0.93		22.48 ± 0.93	
CHD/hBMSC (+)	91.60 ± 23.38	26.94 ± 25.43		61.93 ± 21.83
CHD/hASC (+)	85.22 ± 11.20	9.59 ± 3.56	45.55 ± 12.81	30.08 ± 12.80

*FT* fibrous tissue, *TT* transitional tissue, *HT* hyaline tissue

^a^Col II/hASC (-) and Col II/hBMSC (-): hASC or hBMSC seeded on Col II scaffolds incubated without chondrogenic differentiation medium. Col II/hASC (+) and Col II/hBMSC (+): hASC or hBMSC seeded on Col II scaffolds incubated with chondrogenic differentiation medium. DHC/hASC (-) and DHC/hBMSC (-): hASC or hBMSC seeded on DHC scaffolds incubated without chondrogenic differentiation medium. DHC/hASC (+) and DHC/hBMSC (+): hASC or hBMSC seeded on DHC scaffolds incubated with chondrogenic differentiation medium

**Fig. 7:**
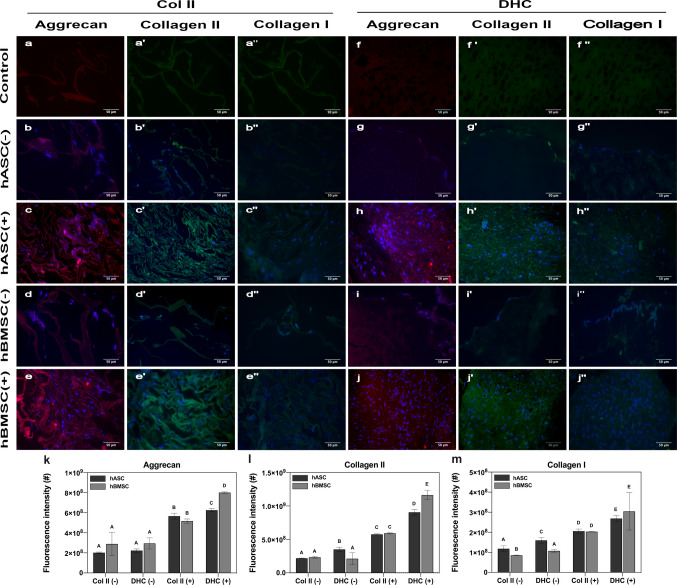
Immunohistochemical analyses. Figure shows aggrecan, collagen type II and type I immunostaining of (a, b, c, d, e – a’, b’, c’, d’, e’ – a’’, b’’, c’’, d’’, e’’) Col II and (f, g, h, i, j – f’, g’, h’, i’, j’ – f’’, g’’, h’’, i’’, j’’) DHC scaffolds seeded with (b-c’’, g-h’’) hASC or (d-e’’, i-j’’) hBMSC and cultured with (+) or without (–) chondrogenic differentiation medium for 28 days. Quantification of the immunofluorescence intensity of (k) aggrecan, (l) collagen II and (m) collagen I. Number of replicas (*n*) = 3; mean ± SD; groups that do not share a letter are significantly different* p* < 0.05. There are no significant differences between samples with the same letter

Representative images of immunohistochemical assays and immunofluorescence quantification are shown in Fig. 7. Autofluorescence was observed in all controls. The autofluorescence detected in the controls with anti-aggrecan antibody is due to traces of proteoglycan in the scaffolds (see “Fourier Transform Infrared Spectrometry (FTIR) (Fig. 7a, f). The controls stained with the anti-collagen II antibody shows the autofluorescence of collagen II (Fig. 7a’, f’). Controls stained with anti-collagen I antibody indicate the existence of cross-reactivity between this antibody and the collagen II present in the scaffolds (Fig. 7a’’, f’’). hASCs or hBMSCs cultured on Col II and DHC scaffolds without chondrogenic differentiation medium barely expressed aggrecan (Fig. 7b, d, g, i), collagen II (Fig. 7b’, d’, g’, i’) and collagen I (Fig. 7b’’, d’’, g’’, i’’). The expression of aggrecan and collagen II—major components of articular cartilage—was clearly observed when the cultures were incubated with chondrogenic differentiation medium (Fig. 7c, c’, e, e’, h, h’, j, j’).

The signal intensity of aggrecan expressed in hASC or BMSC cultured on Col II and DHC scaffolds with DMEM was not significantly different (*p* > 0.05). The same was true for cells seeded on Col II scaffolds that were incubated with chondrogenic differentiation medium. However, there were significant differences (*p* < 0.05) between the signals of hASC and BMSC cultured on DHC scaffolds in the presence of differentiation medium (Fig. 7k). When hASCs or hBMSCs were seeded on Col II scaffolds and incubated with DMEM, no significant differences in the intensity of collagen II signals were observed, but when they were seeded on DHC scaffolds in the presence of the same medium, a significant difference was found. In cultures performed with differentiation medium, no significant differences were observed when these cells were seeded on Col II scaffolds; the opposite was true when these cells were seeded on DHC scaffolds (Fig. 7l).

Significant differences (*p* > 0.05) in the intensity of collagen I immunofluorescence signal were observed when hASCs or BMSCs were seeded on Col II and DHC scaffolds incubated with DMEM, whereas no significant differences (*p* > 0.05) were observed between the cultures incubated with chondrogenic differentiation medium. The intensity of collagen I immunofluorescence signal was different (*p* < 0.05) when hASCs or hBMSCs were seeded on Col II and DHC scaffolds and incubated with DMEM. This did not occur when cultures were performed with chondrogenic differentiation medium (Fig. 7m).

#### Quantitation of angiogenic factors

**Fig. 8:**
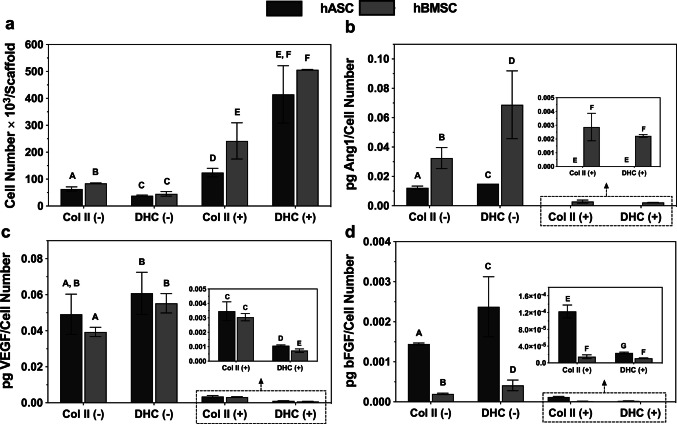
Quantification of cells and angiogenic factors. (**a)** Number of cells in seeded scaffolds at 28 days of culture, (**b)** Angiopoietin-1, **c** VEGF; and (**d)** bFGF concentration in media from 28 day-cultures of hASC or hBMSC seeded in Col II and DHC scaffolds with (+) or without (-) chondrogenic differentiation medium for 28 days. Groups that do not share a letter are significantly different (*p* < 0.05). Number of replicas (*n*) = 3; mean ± SD; the groups that do not share a letter are significantly different *p* < 0.05

Human mesenchymal cells express different factors during differentiation, expression that can be modulated by culture conditions. For example, when cultured on cartilage extracellular matrix (ECM) hydrogels, these cells decreased the secretion of angiogenic factors and increased the expression of chondrocyte markers compared to MSCs cultured in polystyrene flasks (Burnsed et al. 2016). Furthermore, the histomorphometric data from this work show that both the culture medium and the type of scaffold influence de novo cartilage formation. Considering this, we decided to quantify the secretion of angiopoietin-1, VEGF and bFGF in hASCs or hBMSCs cultured on Col II and DHC scaffolds, with or without chondrogenic differentiation medium (Fig. 8). Initially, the number of cells per scaffold was determined to normalize the concentration of factors secreted by cells into the culture medium. Overall, a significantly higher number (*p* < 0.05) of hASCs and hBMSCs was found in cultures grown with chondrogenic differentiation medium than in those grown without (Fig. 8a).

The concentrations of the analyzed factors were in the picogram range. The concentration of angiopoietin-1 was higher in hBMSCs than in hASCs grown with DMEM. When cultures were incubated with chondrogenic differentiation medium, this cytokine was only secreted by hBMSCs seeded on both types of scaffolds. However, its concentrations were lower than those found in DMEM (Fig. 8b). No significant differences were observed between VEGF concentrations detected in hASCs or hBMSCs seeded on Col II (*p* > 0.05) or DHC scaffolds (*p* > 0.05) cultured with DMEM. With the chondrogenic differentiation medium, VEGF concentrations decreased and were not different (*p* > 0.05) when cells were seeded on Col II scaffolds; however, it was higher in hASCs than in hBMSCs cultured on DHC scaffolds (Fig. 8c). With or without chondrogenic differentiation medium, bFGF concentration in hASC cultures was significantly higher (*p* < 0.05) than in hBMSC cultures grown with either scaffold. Interestingly, bFGF secretion decreased significantly (*p* < 0.05) in the presence of chondrogenic differentiation medium, being higher in cells seeded on Col II than in those seeded on DHC scaffolds (Fig. 8d).

## Discussion

In this work, Col II and DHC scaffolds, both made from bovine trachea, were developed using different procedures. Their microstructure, some physicochemical and mechanical properties, and bioactivity were characterized and compared. Furthermore, three angiogenic factors secreted by hASCs and hBMSCs cultured on these scaffolds, with or without chondrogenic differentiation medium, were quantified.

First, a procedure to decellularize hyaline cartilage was established by combining and modifying already published methods (Yang et al. 2010; Gong et al. 2011; Schwarz et al. 2012). The procedure was not time-consuming, inexpensive, and effective, as indicated by the low DNA content (4 ng/mg of tissue) found in the DHC scaffolds, which was lower than the maximum DNA amount (50 ng/mg of tissue) suggested for decellularized tissues (Crapo et al. 2011). In addition to eliminating chondrocytes, it removed the α-gal epitope, ensuring the safety of the decellularized scaffold for human use (Kasravi et al. 2023). The hydroxyproline assay is a gold standard for quantifying total collagen content in a sample. In this study, we used it to indirectly quantify collagen II because this protein represents 95% of the total collagen content of the extracellular matrix of hyaline cartilage (Hu et al. 2024). As demonstrated by the percentage of collagen II after decellularization, this method preserved this protein, suggesting the conservation of the basic microstructure of cartilage.

Unlike the quantifications performed during the biochemical evaluation of the decellularization product, in which the hydroxyproline and GAG contents of the decellularized scaffolds were compared with the contents of these compounds in non-decellularized hyaline cartilage, the quantification of collagen II and GAGs in both types of scaffolds was performed using calibration curves made with known concentrations of hydroxyproline and chondroitin sulfate, respectively. The higher collagen II content in Col II scaffolds than in DHC scaffolds is explained by the fact that the former were prepared with the isolated collagen, which had a very low proteoglycan content, as demonstrated by the results of the ATR-FTIR analysis shown in this work. The higher GAG content in DHC than in Col II scaffolds suggests that decellularization is less effective at removing GAGs than the process used to isolate tracheal collagen II. These data also indicate that the methods used to isolate collagen II and decellularize tracheal hyaline cartilage did not completely remove GAGs.

The reduction in GAG content upon decellularization found in this work may affect the mechanical properties of hyaline cartilage, as its function is to interact with water molecules to resist compression in load-bearing joints (Xu et al. 2025). Despite the low GAG content in the DHC scaffolds detected, the cultured hASCs and hBMSCs synthesized collagen II and GAGs upon incubation with chondrogenic differentiation medium, rendering GAG depletion transient. This phenomenon, previously described by other authors (Li et al. 2019a, b), might have contributed to improving DHC cytocompatibility by allowing its replacement by de novo synthesized hyaline tissue. Our data also support that GAG remnants enhance the cytocompatibility of decellularized scaffolds (Schwarz et al. 2012; Sutherland et al. 2015).

The pore size and porosity of scaffolds are structural characteristics that directly influence the rate of cell proliferation, differentiation, and extracellular matrix synthesis (Yadav et al. 2021). This study found a higher number of hASCs and hBMSCs with rounded nuclear morphology and a higher percentage of extracellular matrix and de novo synthesized hyaline tissue in DHC scaffolds (with smaller pore size and porosity) than in Col II scaffolds (with higher pore size and porosity). While there is no consensus on the ideal pore size and porosity of a scaffold for articular cartilage repair, our histological and histomorphometric findings indicate that the porosity and pore size of DHC scaffolds favored chondrogenesis in the presence of chondrogenic differentiation medium. The small pore size and low porosity of DHC scaffolds may have increased cell condensation and decreased oxygen diffusion, causing hypoxia, parameters that favor cartilage matrix synthesis (Conoscenti et al. 2025).

The LSC of the Col II scaffolds was significantly higher than that of the DHC scaffolds because the larger pore size and porosity of the former allowed them to capture more fluids than the DHC scaffolds. This result confirms that the LSC of the scaffolds depends on their microstructure and hydrophilicity (Nasiri and Mashayekhan 2017; Montazerian et al. 2019). The CA data shown here indicate that both scaffolds were hydrophilic (Huang et al. 2018) and suggest that the lower hydrophilicity of the DHC scaffolds might favor chondrogenesis.

Despite the ζ values of both scaffold types, the adhesion of cultured MSC cells could have been facilitated by cationic proteins in the culture medium adsorbed on the surface of the negatively charged scaffolds (Cámara-Torres et al. 2021). Chondrocytes seeded on negatively charged hydrogels have been shown to synthesize more cartilage extracellular matrix components (collagen II, and aggrecan) than when seeded on positively charged hydrogels (Dadsetan et al. 2011). Furthermore, another work demonstrated that negative charges in collagen hydrogel would enhance chondrogenic differentiation of BMSCs in vitro and in vivo (Yang et al. 2020). In this work, the qualitative and quantitative differences found between the tissue synthesized in each scaffold by hBMSCs and hASCs incubated with chondrogenic differentiation medium were not related to ζ because there were no significant differences between the ζ values of the Col II and DHC scaffolds.

Collagen II scaffolds were cross-linked with genipin to improve their resistance to degradation. The amount of genipin used for cross-linking had already been described in a previous work by our research group (González-Duque et al. 2024). On the contrary, DHC scaffolds were not cross-linked because decellularized scaffolds perform better in vivo when uncross-linked. Indeed, cross-linking of decellularized scaffolds can lead to chronic foreign body response, fibrotic encapsulation, and poor in vivo outcomes. Cross-linking has been reviewed to alter the bioactivity of decellularized cartilage scaffolds and decrease their chondroinductivity by modifying their intrinsic characteristics. Cross-linking also prevents the release of degradation products from cartilage-derived scaffolds that promote chondrogenic differentiation (Burnsed et al. 2016; Krishtul et al. 2020; Rowland et al. 2018). The rapid degradation of DHC scaffolds observed in our study could have contributed to greater chondrogenic differentiation in the cultures in which they were used, especially those seeded with hBMSCs.

In the in vitro enzymatic degradation assay, the DHC scaffold was almost completely degraded at 8 h and its degradation was greater than that of the Col II scaffold 2 h after the start of the test. This may be explained by the impact of decellularization on the composition and microstructure of the cartilage extracellular matrix (ECM) and because, unlike the Col II scaffolds, the DHC scaffolds were not cross-linked.

The observed in vitro degradation rate of the DHC scaffold appears too rapid to be synchronized with new tissue synthesis. However, when hBMSCs and hASCs were cultured on these scaffolds for 28 days with chondrogenic differentiation medium, the formation of high percentages of hyaline tissue was observed (see Table 3). This indicates that the DHC scaffold, by interacting with cells during their chondrogenic differentiation, is being replaced as it degrades. Furthermore, the percentage of hyaline tissue was higher when hBMSCs and hASCs were cultured on DHC scaffolds than when they were cultured on Col II scaffolds, suggesting that the enzymatic degradation rates of scaffolds differentially influence chondrogenesis. The rapid degradation of scaffolds has been reported to improve their chondrogenic performance in vitro (Sarem et al. 2018) and promote higher quality tissue synthesis in vivo than in vitro (Shah et al. 2018). Furthermore, non-crosslinked decellularized cartilage scaffolds degrade rapidly in vitro compared to crosslinked ones (Pinheiro et al. 2016; Elder et al. 2017).

This work showed that DHC scaffolds, with smaller pore sizes and porosity than Col II scaffolds, exhibit the best mechanical properties confirming that these depend on the scaffold’s microstructure (Yadav et al. 2021). Our results are consistent with those reported by other authors, who show an inverse relationship between porosity and pore size, and the in vitro mechanical properties of scaffolds (Nasiri and Mashayekhan 2017).

The percentage of hyaline tissue synthesized by cells cultured on Col II and DHC scaffolds with chondrogenic differentiation medium was observed to increase as scaffold contraction increased. This suggests a correlation between the percentage of extracellular matrix and tissue types synthesized with scaffold contraction. Several studies have shown that scaffold contraction provides a high-density cellular environment that favors extracellular matrix biosynthesis by mimicking the cell condensation phenomenon required for chondrogenesis (Irawan et al. 2018; Li et al. 2019a, b).

Histomorphometric analyses confirmed the presence of different tissue types within the same scaffold. hBMSCs cultured on DHC scaffolds with chondrogenic differentiation medium synthesized a high percentage of hyaline tissue and a low percentage of fibrous tissue, without forming transitional tissue. Under the same culture conditions, hASCs formed a higher percentage of transitional tissue than hyaline tissue. Finally, both mesenchymal cell types cultured on Col II scaffolds with chondrogenic differentiation medium synthesized more transitional tissue than hyaline and fibrous tissue, the latter located at the periphery of the scaffolds. Previous studies have found that mesenchymal stem cells at the periphery of hyaluronic acid scaffolds express smooth muscle actin (α-SMA) and are responsible for mediating scaffold contraction (Toh et al. 2012). The heterogeneity of the de novo tissue synthesized when the cultures were incubated with the chondrogenic differentiation medium may be related to the difference in the characteristics of the scaffolds and to the phenotypic and functional variations of the mesenchymal stem cells (Li et al. 2023).

Overall, the percentages of extracellular matrix and hyaline tissue observed in cultures incubated with chondrogenic differentiation medium confirm that chondrogenic factors are necessary for the formation of hyaline cartilage (Zhao et al. 2022).

Regardless of whether the cultures were grown under chondrogenic differentiation conditions or not, they produced transitional tissue, except for hBMSC cultured on DHC scaffolds in the presence of chondrogenic differentiation medium, which primarily synthesized hyaline tissue. This result suggests that these scaffolds, with characteristics similar to those of native cartilage, possess intrinsic chondrogenic potential (Kim et al. 2019). It also suggests that cells require additional stimuli to those provided by decellularized cartilage to induce hyaline tissue formation.

This study revealed that chondrogenic differentiation medium favored hBMSC differentiation over hASC differentiation, as indicated by the percentages of extracellular matrix (ECM) and tissue types formed on the scaffolds. This result indicates that specific chondrogenic factors are required to induce chondrogenesis in hASC cultures to the same extent as that observed in hBMSCs cultured on DHC and Col II scaffolds.

The loss of the avascular state of cartilage affects its proper function (Pei et al. 2022). Our evaluation of three angiogenic factors in the culture media showed an inverse relationship between chondrogenesis and the in vitro secretion of angiopoietin-1, VEGF, and bFGF. This result is consistent with previous research demonstrating that undifferentiated mesenchymal stem cells seeded in hydrogels secrete angiogenic factors, the concentration of which decreases as chondrogenic differentiation occurs (Bara et al. 2014). Furthermore, it is important because the decreased secretion of angiogenic factors by cells cultured on scaffolds can impede their mineralization and, consequently, the likelihood of failure of these artificial substitutes (Burnsed et al. 2016).

The decrease in the angiogenic factors evaluated could be due to antiangiogenic factors secreted by cells synthesizing new tissue in the presence of a chondrogenic differentiation medium. It is also possible that the lower secretion of these three factors in cultures using DHC scaffolds as a substrate is due to the preservation of the antiangiogenic factors present in hyaline cartilage after decellularization (Pei et al. 2022).

## Conclusions

In this work, we established a rapid and economical procedure for decellularizing bovine tracheal hyaline cartilage. We also produced and characterized Col II and DHC scaffolds, which showed microstructural, physicochemical, and mechanical differences that affect their bioactivity. Under culture conditions that promoted chondrogenic differentiation, the percentage of de novo synthesized ECM and hyaline-like tissue was higher in cultures grown on DHC scaffolds than in those grown on Col II scaffolds, especially when hBMSCs were used. The secretion of angiopoietin-1, VEGF, and bFGF was downregulated in cultures where mainly hyaline-like or transitional tissue was formed. Furthermore, the concentration of these angiogenic factors was lower in DHC cultures than in Col II cultures. Overall, data shown support that the type of mesenchymal stem cell and the substrate used to grow them with chondrogenic medium, differentially promote chondrogenic differentiation. Furthermore, they demonstrate the potential of DHC scaffolds to promote de novo synthesis of hyaline cartilage.

## Data Availability

Data is provided within the manuscript. Any additional information will be provided upon request.
